# Dental structure and tooth attachment modes in the common fangtooth *Anoplogaster cornuta* (Valenciennes, 1833) (Actinopterygii; Trachichthyiformes; Anoplogastridae)

**DOI:** 10.1371/journal.pone.0272860

**Published:** 2022-08-12

**Authors:** Horst Kierdorf, Uwe Kierdorf, Hartmut Greven, Günter Clemen

**Affiliations:** 1 Department of Biology, University of Hildesheim, Hildesheim, Germany; 2 Department of Biology, University of Düsseldorf, Düsseldorf, Germany; 3 Doornbeckeweg 17, Münster, Germany; Universiteit Gent, BELGIUM

## Abstract

We studied the structure and attachment modes of the teeth of adult *Anoplogaster cornuta* using light- and scanning-electron microscopic techniques. All teeth were monocuspid, composed solely of orthodentin, and lacked a covering enameloid cap. Fourteen teeth were present in the oral jaws, with three teeth each on the left and right premaxilla and four teeth each on the left and right dentary. The anteriormost premaxillary and dentary teeth were considerably larger than the more posteriorly located ones. The oral jaw teeth were transparent, non-depressible and firmly ankylosed to their respective dentigerous bone by a largely anosteocytic bone of attachment. No evidence for replacement of the large oral jaw teeth was found in the analyzed adult specimens. The bone of attachment exhibited lower calcium and phosphorus concentrations and a higher Ca/P ratio than the orthodentin. The connection between dentinal tooth shaft and bone of attachment was stabilized by a collar of mineralized collagen fibers. In contrast to the oral jaw teeth, the pharyngeal teeth exhibited a ring-like fibrous attachment to their supporting bones. This mode of attachment provides the teeth with some lateral mobility and allows their depression relative to their supporting bones, which may facilitate intra-pharyngeal prey transport. In contrast, a firm ankylosis was observed in numerous small teeth located on the branchial arches. The function of these teeth is presumably to increase the tightness of the pharyngeal basket and thereby the retention of small prey items in a species living in a habitat with only sparse food supply. Our findings corroborate earlier statements on the tooth attachment modes of the oral jaw teeth of *Anoplogaster cornuta*, but provide new findings for the attachment modes of pharyngeal teeth in this species.

## Introduction

**Fig 1:**
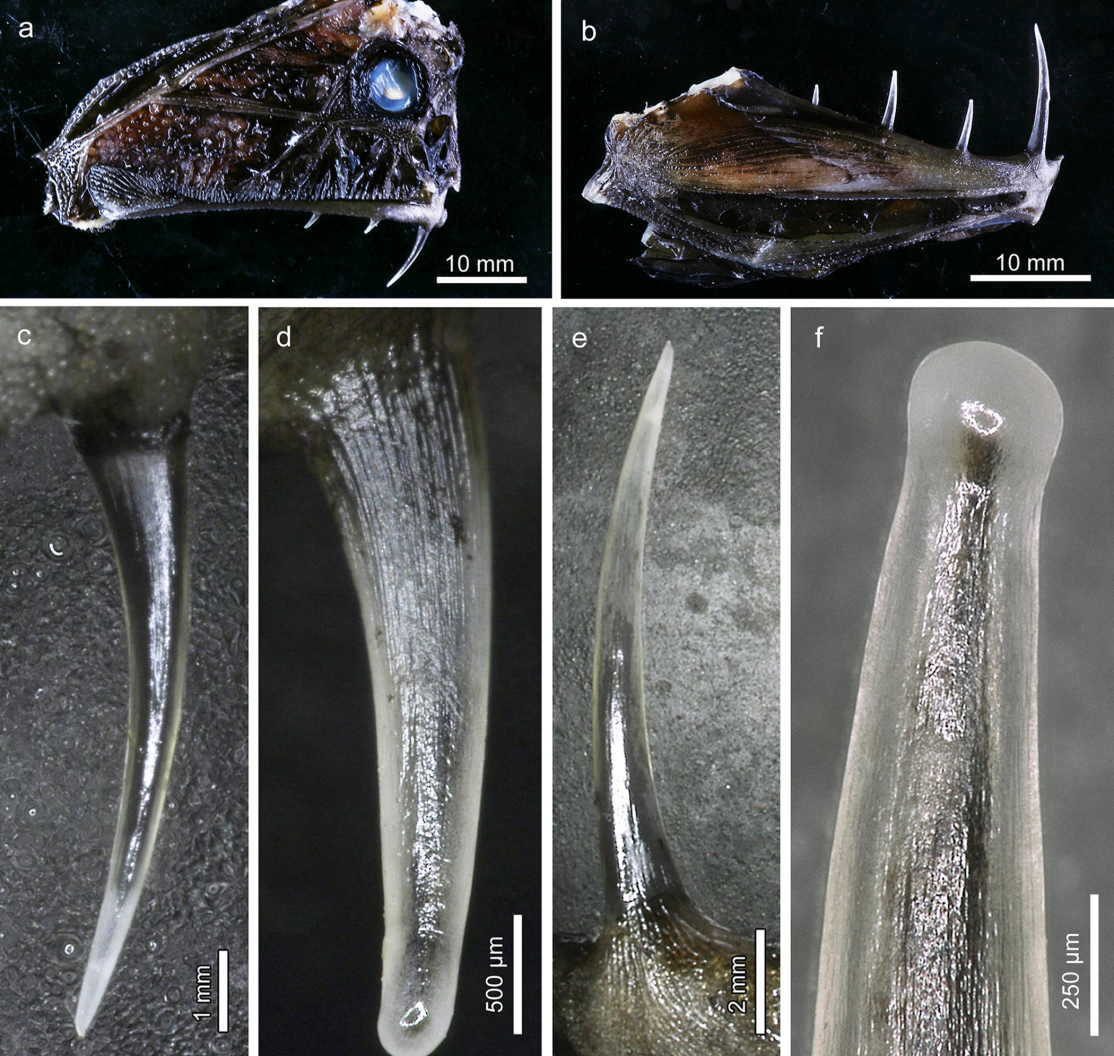
Oral jaw teeth in *Anoplogaster cornuta*. **(a)** Right lateral view of head showing the three teeth on the premaxilla. **(b)** Right lateral view of the lower jaw showing the four teeth on the dentary. **(c)** Right first premaxillary tooth, lateral view. Note transparency of the dentin and pointed tooth tip. **(d)** Right third premaxillary tooth, lateral view. Note rounded tooth tip. **(e)** Left first dentary tooth, lateral view. Note pointed tooth tip. **(f)** Rounded, knob-like tip of right second dentary tooth, medial view. Due to the transparency of the dentin the extension of the pulp cavity is visible.

The family Anoplogastridae belongs to the order Trachichthyiformes within the Acanthomorpha. The latter is a large clade of advanced teleosts (Neoteleostei) within the Actinopterygii (ray-finned fishes) [1]. The Anoplogastridae comprise a single genus (*Anoplogaster*) of bathypelagic to mesopelagic fishes with a worldwide distribution in tropical to temperate and subarctic waters [1,2]. The two species within the genus are the common or longhorn fangtooth (or sabretooth) (*Anoplogaster cornuta*) and the shorthorn fangtooth (or sabretooth) (*Anoplogaster brachycera*). Juveniles of the former species possess long temporal and preopercular spines that are missing in the latter [3]. The common name of the genus refers to the elongate fanglike teeth that project out of the mouth of adults (Fig 1A and 1B). Relative to its head size, which accounts for about one-third of the maximum adult body length of about 16 to 17cm, *Anoplogaster cornuta* possesses the largest teeth of any marine fish [2]. The long anterior fang teeth of the oral jaws prevent adult individuals from completely closing their mouth, with the fangs of the lower jaw sliding into pockets in the roof of the mouth when it is partially closed [4].

Juvenile and adult individuals of *Anoplogaster* spp. are morphologically so dissimilar that they were long considered different species [3]. While adults are commonly captured at depths between 75 and 5000 m, juveniles are found between 45 and 3000 m and larvae at shallow depths [2]. Juveniles possess only small teeth and mainly feed on small crustaceans [3]. The large anterior fang-like teeth start to develop in the lower jaw when the animals reach about 2 cm body length, and in the upper jaw at about 5.3 cm body length, and juveniles begin to resemble adults when attaining a body length of about 8 cm [3]. Adult individuals mainly feed on fish and shrimps that are typically swallowed whole. Prey size of adults can reach one-third of their body size, necessitating a reverse gill ventilation during feeding [2,5].

Observation on the feeding behavior of adult specimens in captivity revealed that they swim slowly by sculling with their pectoral fins until they bump into a prey, whose touch on their mouth region elicits a feeding response [6]. It was further reported that, in addition to mechanical stimulation, contact chemoreception seems to play an important role in triggering the feeding response of *Anoplogaster cornuta*. These authors suggested that contact chemoreception may serve as a ‘close-range’ system for detecting prey items once the fangtooth has approached potential prey using other sensory system (e.g. the lateral line system) in the dark deep-sea environment [6].

The highly variably feeding modes of teleost fishes and the associated manifold mechanical demands on their teeth are reflected by a huge diversity of tooth forms and different modes of attachment of the teeth to their supporting (dentigerous) bones [4,7,8]. These attachment modes range from a firm fixation (ankylosis) by a bridging mineralized tissue located between the dentinal tooth base and the dentigerous bone to specialized types of fibrous attachment that allow for a certain mobility of the teeth against their supporting jaw bones [4,7]. Most authors consider ankylosis between tooth and dentigerous bone as the ancestral character state [4,7,8]. Accordingly, fibrous attachment modes are thought to represent a derived condition, brought about by a cessation of mineralization before complete ankylosis between tooth base and dentigerous bone is achieved.

Fink [7] distinguished four major types of tooth attachment in actinopterygian fishes, with Types 1 and 2 being the more frequent ones. In Type 1, the teeth are rigidly ankylosed to the underlying dentigerous bone by a mineralized tissue, referred to as attachment bone or bone of attachment [4,8]. In the Type 2 mode of attachment, a dividing zone at the base of the teeth remains completely unmineralized and the dentinal tooth base is connected to the underlying bone of attachment by a ringlike collagenous ligament, which allows some movement of the tooth against the bone. Types 3 and 4 of Fink’s classification constitute so-called hinged types of tooth attachment. In Type 3 (anterior-hinged attachment), the mineralization of the tooth base extends into the anterior region of the fibrous attachment zone while posteriorly an unmineralized collagenous attachment is present between the tooth base and the bone of attachment. The mineralized anterior connection forms a hinge point with an anterior axis of rotation. In Type 4 (posterior-hinged attachment), the collagenous connection between tooth base and bone of attachment is missing anteriorly; instead, a gap (dividing zone) is present between tooth base and bone at this location. An unmineralized collagenous connection is present only posteriorly, providing a hinge with a posterior axis of rotation [4,7].

The nature of the bone of attachment, i.e., its classification as either dentin, bone, or an intermediate type of mineralized tissue, has long been debated (for a review see [8]). Recent studies in zebrafish (*Danio rerio*) demonstrated that the cells that deposit the bone of attachment express genetic markers of osteoblasts but not of odontoblasts [8]. In zebrafish, the bone of attachment exhibits histologic characteristics that are intermediate between those of dentin and bone, and Rosa et al. [8] have therefore proposed the term “dentinous bone” for the bone of attachment.

At present, only little information is available on the structure and the attachment modes of the teeth of *Anoplogaster cornuta*. In his review, Fink [7] listed a Type 1 attachment mode for the oral jaw teeth and a Type 4 attachment mode for the pharyngeal teeth of adult individuals from this species. The present study provides novel information on dental structure and mineralization, and on the tooth attachment modes in adult *Anoplogaster cornuta*.

## Materials and methods

### Specimens

Two adult specimens of *Anoplogaster cornuta* (total length of 12.0 and 13.5 cm, respectively) were caught in 2002 at depths between 680 and 708 meters during an expedition (M244) to the Irmiger and Greenland Sea (North Atlantic Ocean) organized by the Institute of Marine Biology, Alfred-Wegener Institute, Bremerhaven (Germany). Both specimens were frozen *in toto* on board and given to one of the authors (HG) for study.

### Specimen preparation, image acquisition and elemental (Ca, P) analysis

Upon arrival in the lab, the frozen specimens were thawed and fixed in 70% ethanol and their heads were removed. Following fixation, one head was cleared and stained with Alcian Blue 8GX (cartilage) and Alizarin Red S (bone), using an established method [9,10]. The stained and the unstained head were photographed with a digital camera (Canon EOS 80D) equipped with a macro lens. Subsequently, the jaws were dissected free and the upper and lower oral jaw teeth as well as the tooth-bearing structures of the oropharynx were photographed at higher magnifications under reflected light with a digital microscope (Keyence VHX-500F) using high-performance lenses of, respectively, 5 to 50 times and 20 to 200 times magnification. The oral jaws and other tooth-bearing structures were then freed from most of the adhering soft tissue by manual dissection and, partly, also by digestion with an enzyme solution (Enzyrim OSA; Bauer, Fehraltorf, Switzerland). Following drying at room temperature, the oral jaw teeth and their supporting bones (premaxilla and dentary) were examined uncoated in a scanning electron microscope (SEM) (Zeiss EVO MA 15) operated at 20 keV accelerating voltage in a low vacuum mode, using a high-definition five-segment backscatter electron (BSE) detector.

Subsequently, individual teeth together with portions of the dentigerous bone were dissected from the jaws and embedded in epoxy resin (Biodur E 12, Biodur Products, Heidelberg, Germany). The resulting blocks were then cut with the section plane running either through the tip of the respective teeth (longitudinal sections) or horizontally at different heights through the tip and shaft areas of the teeth (transverse sections). In case of the longitudinal sections, care was taken to include both the tooth tip and the transition zone between the basal tooth shaft and the dentigerous bone in the section plane. For SEM-BSE imaging of the resulting block faces, the cut surfaces were smoothed and polished by hand using a series of silicon carbide papers (up to grit 4000), followed by a final polishing step on a motorized polisher (Labopol 5, Struers, Ballerup, Denmark) using first a diamond suspension of 3 μm particle size and subsequently an alumina slurry of 0.3 μm particle size. The uncoated samples were studied in the SEM as described, with the polished surface oriented perpendicular to the primary electron beam. Gray level variation in the captured BSE-images of the scanned surfaces reflects changes in local mineral content, with brighter gray levels characterizing areas of higher, and darker gray levels areas of lower mineral content [11,12].

In addition, scanning electron microscope-energy dispersive X-ray spectrometry (SEM-EDS) was conducted on the polished block surfaces of a left and a right first dentary tooth. Line scans of calcium (Ca) and phosphorus (P) distribution and multipoint standardless analysis of Ca and P concentrations were performed in the Zeiss Evo Ma 15 SEM with a Bruker XFlash 410-M detector (Bruker, Billerica, MA, USA). In the latter case, eight point measurements each were taken in the dentin and the underlying bone of attachment. The acquired data were processed with the software package Esprit (Bruker).

As the data satisfied the assumptions of normality (Shapiro-Wilk-test) and homoscedasticity (Levene-test), differences in Ca and P concentrations and Ca/P ratio between dentin and bone of attachment were tested for significance using unpaired t-tests.

Ground sections (thickness of about 50 μm) of the epoxy resin embedded specimens were prepared with a previously described grinding and polishing technique [13]. The ground sections were examined in an Axio Imager 2 microscope (Zeiss, Oberkochen, Germany) using either plain transmitted light, transmitted light with phase-contrast enhancement, or circularly polarized transmitted light.

Following the enzymatic digestion of soft tissues, the pharyngeal teeth and their supporting bones were also studied uncoated by BSE imaging in the SEM. One pharyngeal tooth and its underlying dentigerous bone were split open with a scalpel to reveal their inner structure.

## Results

### Oral jaw teeth

A full set of oral jaw teeth in adult *Anoplogaster cornuta* comprised fourteen widely spaced monocuspid teeth that were all slightly recurved in posterior direction. Three teeth each were present in the left and right premaxilla, and four teeth each in the left and right dentary (Fig 1A and 1B). The first (most anteriorly located) premaxillary and dentary teeth were markedly longer than the more posterior ones and exhibited a pointed tip (Fig 1A–1C and 1E). The length of the anteriormost teeth ranged between 10 and 11 mm in the lower and between 8 and 9 mm in the upper jaw. The further posteriorly located teeth were considerably shorter, with lengths ranging between 2.2 mm (fourth dentary tooth) and 4.9 mm (third dentary tooth), and exhibited more rounded, sometimes knob-like tips (Fig 1A, 1B, 1D and 1F). The surface of all oral jaw teeth was characterized by a fine longitudinal corrugation that extended from the base of the teeth almost to their tips. The teeth were translucent so that the extension of the dark-appearing dental pulp was readily discernible on external inspection (Fig 1C–1F).

**Fig 2:**
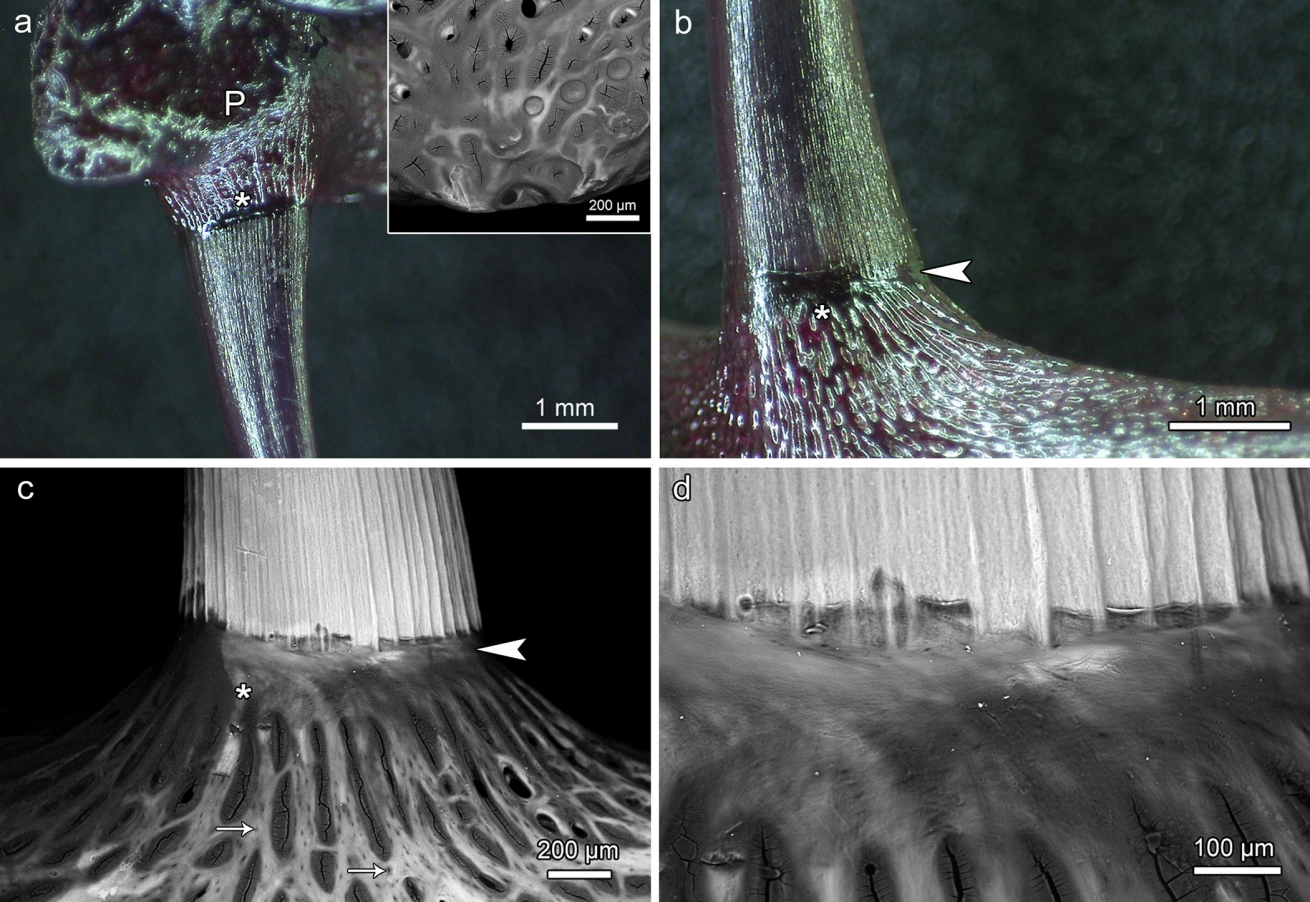
Oral jaw teeth and supporting bone in *Anoplogaster cornuta*. **(a)** Basal portion of right first premaxillary tooth, bone of attachment (asterisk) and premaxillary bone (P), medial view. The cleared and stained specimen shows intense staining of dentin and bone by alizarin red S. Insert: SEM-BSE image of the empty socket of the second premaxillary tooth, lateral view. **(b)** Transition zone between basal portion of left first dentary tooth and bone of attachment (asterisk). Arrowhead: Collar of mineralized collagen fibers. **(c)** SEM-BSE image of transition zone between basal portion of left second dentary tooth and bone of attachment (asterisk), lateral view. Note regular longitudinal corrugation of the tooth surface, collar of mineralized collagen fibers (arrowhead), and presence of numerous osteocyte lacunae (arrows) in the supporting dentary bone compared to lack of such lacunae in the bone of attachment. **(d)** Higher magnification of the transition zone between basal tooth portion and bone of attachment with collar of mineralized collagen fibers.

**Fig 3:**
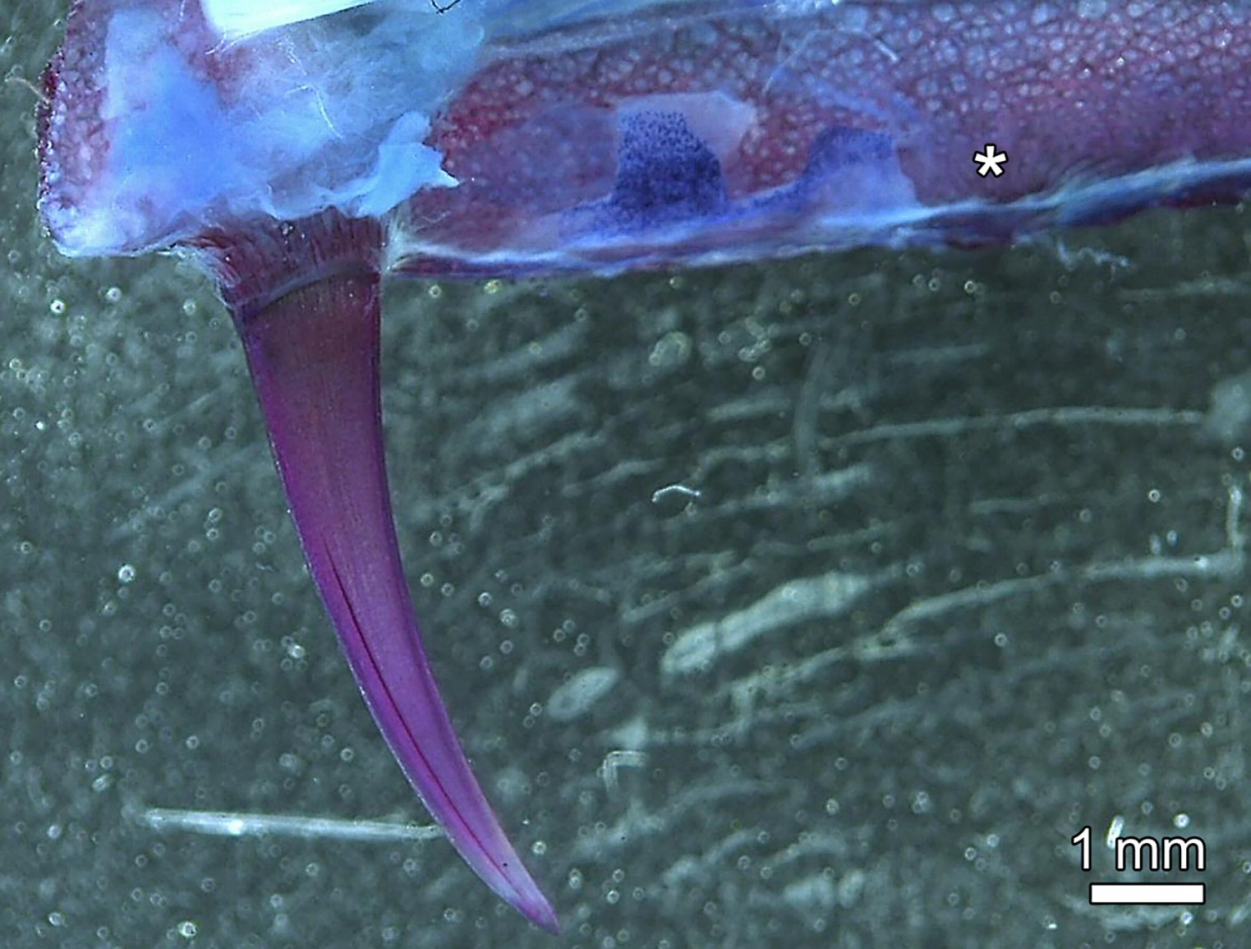
Anterior portion of right premaxilla of *Anoplogaster cornuta*, cleared and stained (Alizarin red S, alcian blue 8 GX) specimen, medial view. No replacement tooth is present at the empty locus (asterisk) of the second premaxillary tooth.

The oral jaw teeth were attached to bony sockets that protruded from the supporting (dentigerous) jaw bones. The jaw bones and the bony sockets exhibited a mesh-like trabecular architecture (Figs 2A–2C and 3). Macroscopic and microscopic inspection revealed that the bony tooth sockets consisted of a proximal portion with numerous osteocyte lacunae and a distal portion largely devoid of osteocyte lacunae (Fig 2C). The proximal portion was diagnosed as part of the dentigerous bone, the distal portion as the bone of attachment. Externally, the tooth base and the adjacent bone of attachment were covered by a collar of mineralized collagen fibers (Fig 2B–2D). In one specimen, the left second premaxillary tooth had been lost, leaving a bare bony tooth socket (Fig 2A, insert and Fig 3). At this locus, as in the premaxillae and dentaries in general, no incoming replacement teeth were found. Also no signs of resorption at the base of the functional oral jaw teeth were observed.

**Fig 4:**
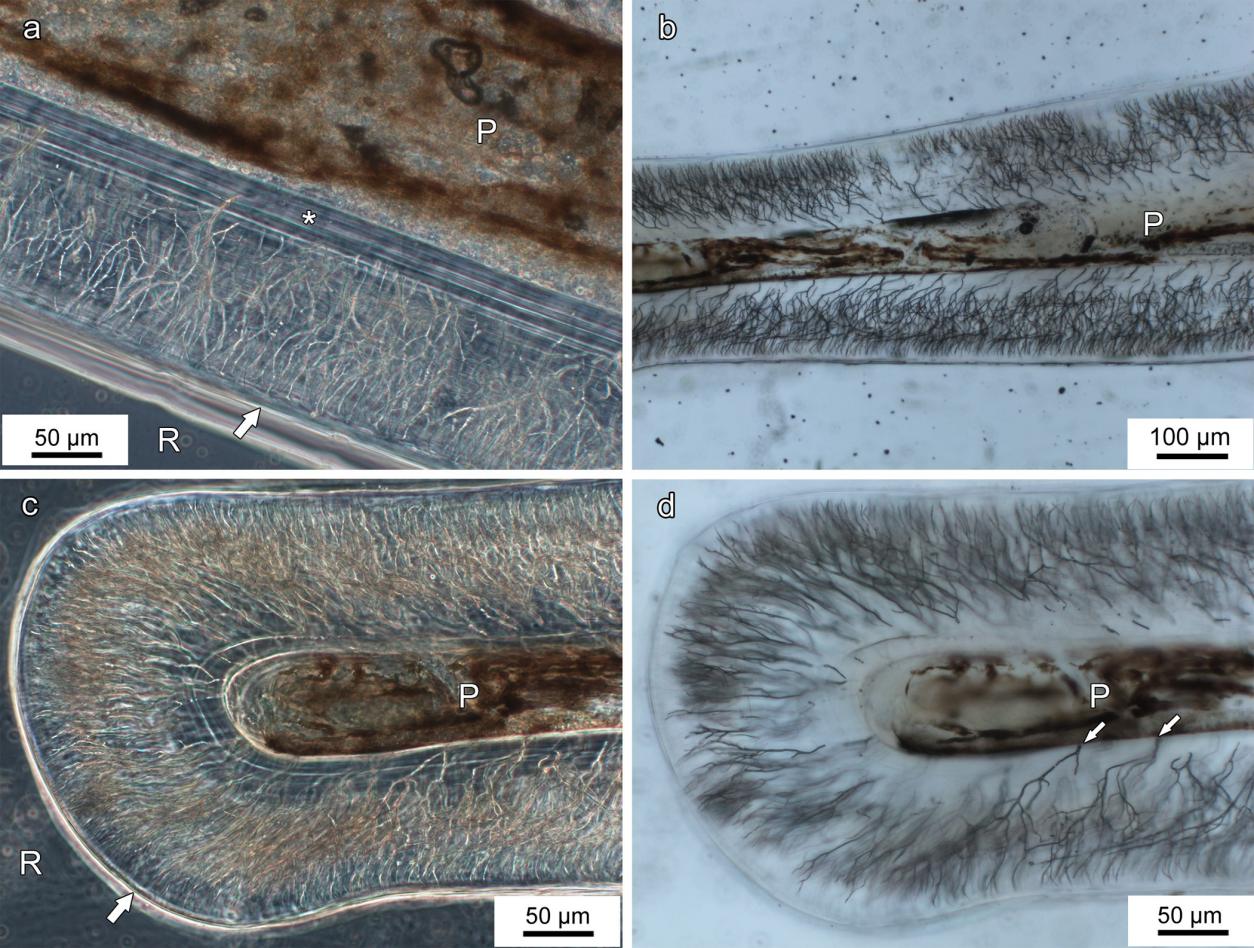
Light micrographs of longitudinal ground sections of lower oral jaw teeth of *Anoplogaster cornuta*. **(a)** Anterior wall of the shaft of the left first dentary tooth showing intense ramification of tubules in central dentin, and inner (juxtapulpal) dentinal zone (asterisk) with only few tubules. P: Pulp cavity. Arrow points to the tooth surface. A cleft between the tooth surface and the embedding resin (R) appears as a bright zone. Transmitted light with phase-contrast. **(b) S**haft of left third dentary tooth showing extension and ramification of dentinal tubules, tooth tip to left of image. P: Pulp cavity. Normal transmitted light. **(c)** Tip of left third dentary tooth showing ramification of dentinal tubules in central dentin. Note lack of an enameloid cap. P: Pulp cavity. Arrow points to tooth surface. A cleft between tooth surface and embedding resin (R) appears as a bright zone. Transmitted light with phase-contrast. **(d)** Tip of left third dentary tooth showing that the dentinal tubules typically cannot be traced up to the tooth surface. Note lack of an enameloid cap. Arrows: Openings of dentinal tubules at the pulpal border. P: Pulp cavity. Normal transmitted light.

**Fig 5:**
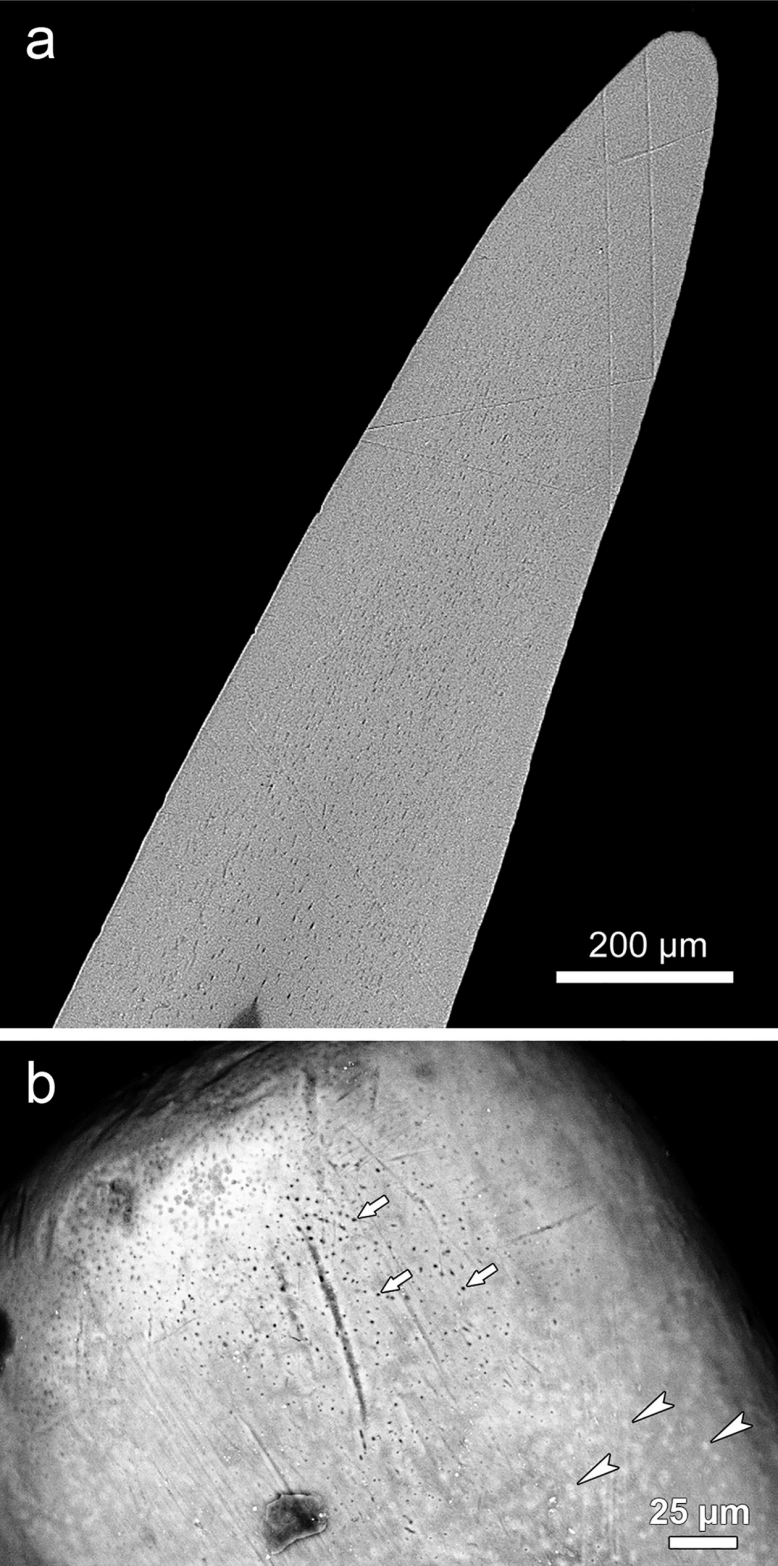
Tip region of oral jaw teeth. **(a)** SEM-BSE image of polished longitudinal section of left first dentary tooth. Note uniform gray level (indicative of homogeneous mineralization) of the dentin and lack of an enameloid cap. **(b)** SEM-BSE image of the surface of a right third dentary tooth, lingual view. At the tooth tip, dentinal tubules have been exposed by wear (arrows). In a less worn area slightly more proximally, the dentinal tubules are occluded by a tissue (arrowheads) that appears brighter (i.e., more mineralized) than the surrounding dentin.

Inspection of ground sections through the oral jaw teeth revealed that they were completely composed of orthodentin that exhibited numerous dentinal tubules (Fig 4A–4D). A cap of hypermineralized tissue (enameloid) overlying the dentin of the tooth tip was not discernible in the ground sections (Fig 4A–4D) and SEM-BSE images (Fig 5A). In the inner (juxtapulpal) dentin portion, tubule density was much lower than in more central dentin portions, where a pronounced tubular branching was observed (Fig 4A–4D). In the ground sections, the dentinal tubules could typically not be traced up to the tooth surface.

**Fig 6:**
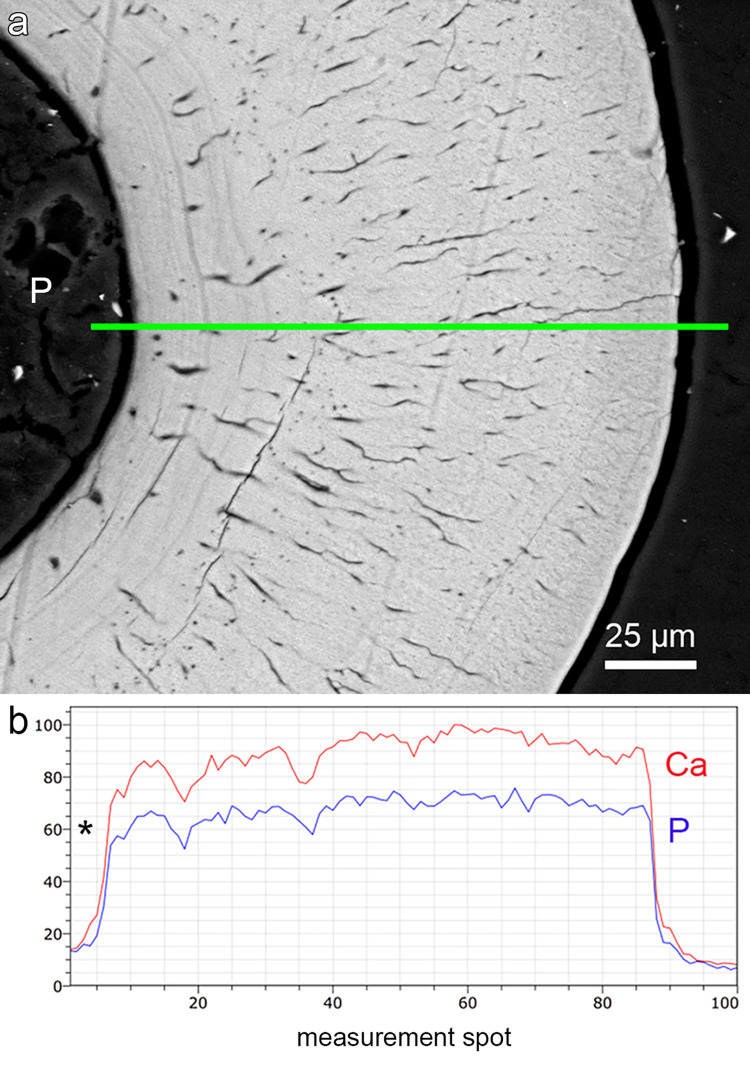
Transverse section through the tip region of a right first dentary tooth of *Anoplogaster cornuta*. **(a)** SEM-BSE image showing uniform gray level distribution (indicative of homogeneous mineralization) of the dentin. The position of the EDS line profile is indicated by the green line. P: Pulp cavity. **(b)** SEM-EDS line profile showing variation of calcium (Ca) and phosphorus (P) concentrations (relative values) across the dentinal wall. An enameloid layer with higher Ca and P concentrations is not present. Asterisk: Pulp cavity.

SEM-BSE images of polished surfaces of longitudinally and transversely sectioned tooth tips showed a uniform gray level of the dental hard tissue, indicative of a largely homogeneous degree of mineralization (Figs 5A and 6). A bright (hypermineralized) surface layer indicative of the presence of enameloid was missing. SEM-BSE imaging of the surface of tooth tips demonstrated that the lumina of the tubules in the outermost dentin were occluded by a mineralized tissue (Fig 5B). This mineralized tissue appeared slightly brighter than the surrounding dentin, thereby indicating a higher degree of mineralization. However, in worn tip areas, openings of the dentinal tubules onto the tooth surface were sometimes discernible (Fig 5B). This indicates that occlusion of the dentinal tubules had occurred only in the outermost dentin. The walls of the pulp cavity were smooth and did not exhibit the dentin pleats characteristic for plicidentin (Fig 4A–4D). A transverse section through the base of a jaw tooth (about 1 cm cuspal to the junction with the bone of attachment) demonstrated that the longitudinal corrugation of the tooth shaft was due to a wavelike undulation of the tooth surface only (S1 Fig).

**Fig 7:**
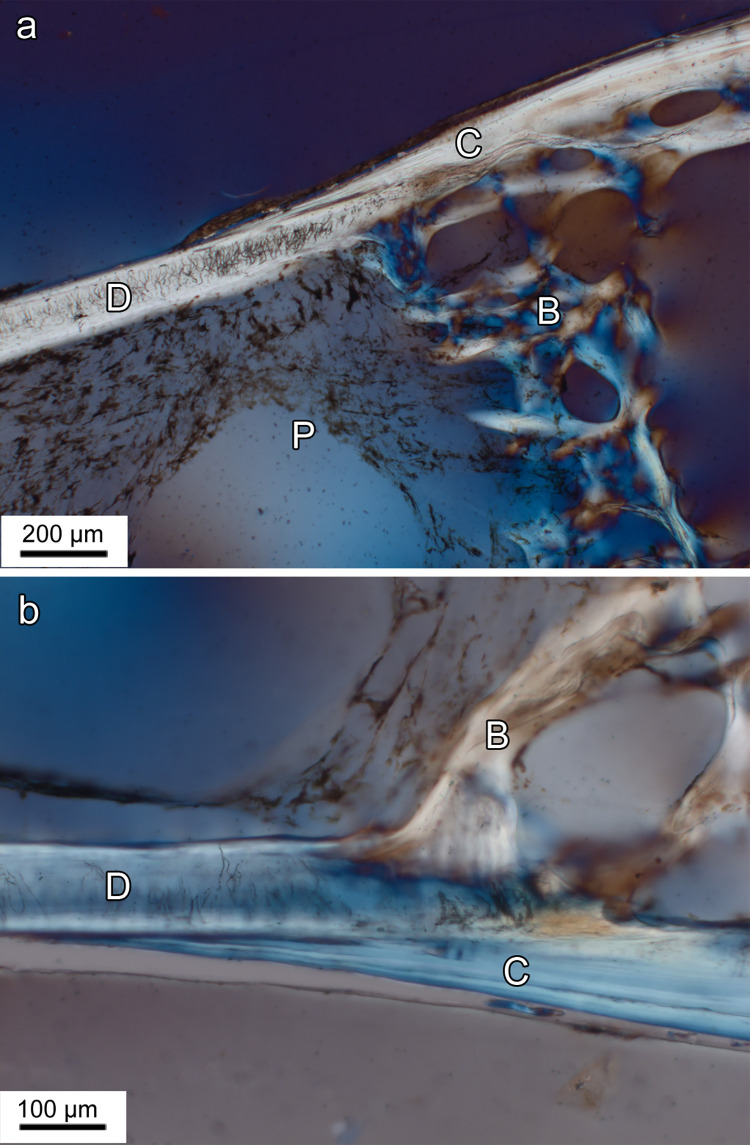
Light micrographs of longitudinal ground sections showing basal portion of lower oral jaw teeth and bone of attachment in *Anoplogaster cornuta*. **(a)** Left first dentary tooth. B: Bone of attachment; C: Collar of mineralized collagen fibers; D: Dentin; P: Pulp cavity. Posterior to top of image. Circularly polarized transmitted light. **(b)** Higher magnification of transition zone between basal anterior dentinal tooth shaft (D) and bone of attachment (B) of left first dentary tooth. C: Collar of mineralized collagen fibers. Circularly polarized transmitted light.

**Fig 8:**
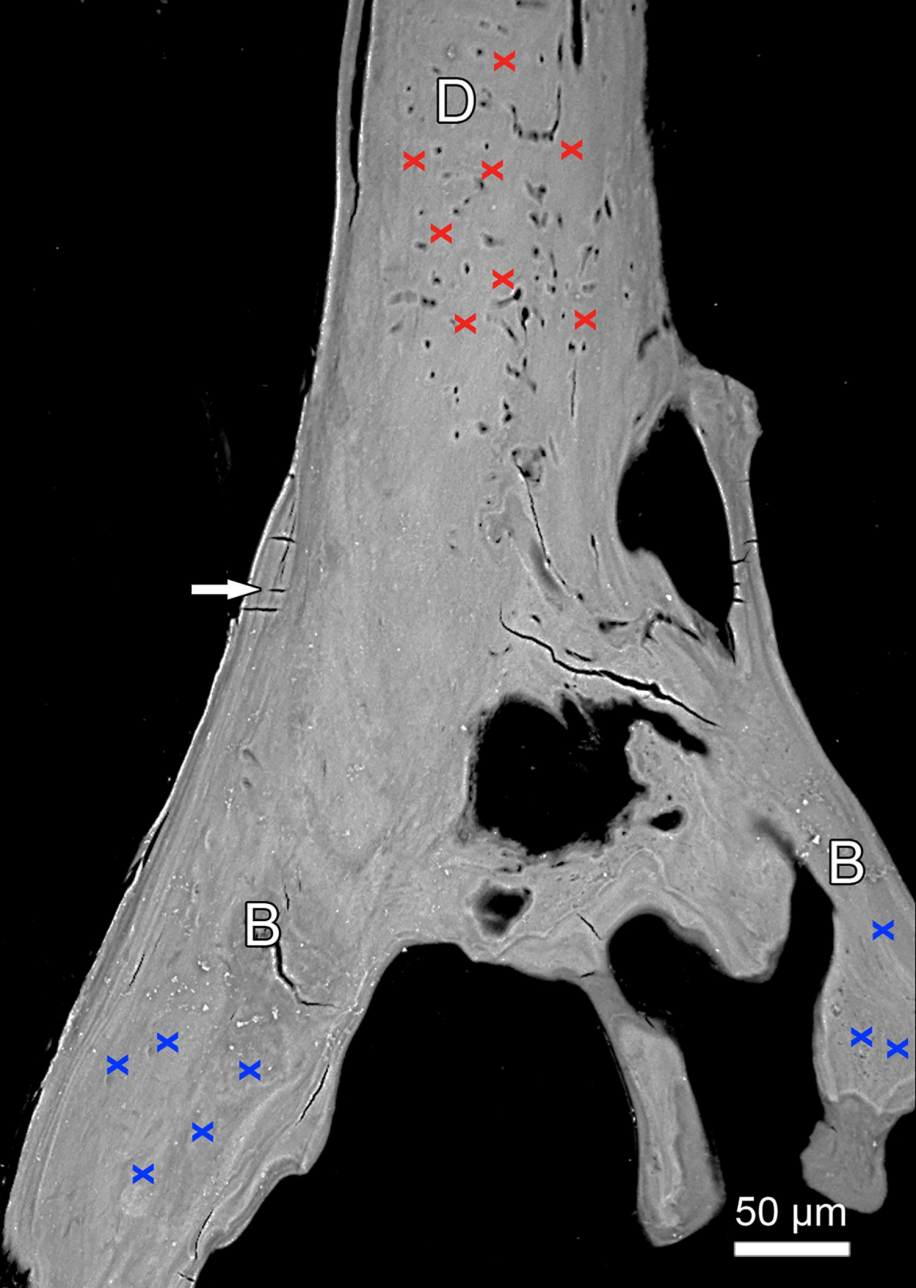
SEM-BSE image of polished longitudinal section through basal portion of left first dentary tooth and adjacent bone of attachment. Location of SEM-EDS point measurement spots is indicated by red crosses in the dentin (D) and blue crosses in the bone of attachment (B). Note lack of osteocyte lacunae in the bone of attachment. Arrow: Mineralized collagen fibers.

In the oral jaw teeth, the dentinal tooth shaft was directly continuous with a proximally adjacent, largely acellular (anosteocytic) bone of attachment (Figs 7A, 7B and 8) that firmly ankylosed the tooth to the underlying supporting (dentigerous) bone. Light and scanning-electron microscopic analysis showed that the dentinal tooth base and the adjacent bone of attachment were overlain by a sleeve-like collar of mineralized collagen fibers that gradually tapered out in cuspal direction (Figs 7A, 7B and 8). This collar could already be identified on macroscopic inspection (Fig 2B–2D). Beneath the central pulp space, the bone of attachment formed a meshwork of bony trabeculae (Fig 7A).

SEM-EDS analysis revealed significantly higher Ca and P concentrations of the dentin compared to the bone of attachment (Table 1). The dentin further had a lower Ca/P ratio than the bone of attachment. A uniform degree of dentin mineralization across the tooth shaft could be demonstrated by horizontally oriented SEM EDS line scans running from the dentin-pulp-interface to the tooth surface (Figs 6 and S1). The slightly brighter outer rim visible on the SEM-BSE image along parts of the tooth surface (Fig 6A) showed a lower Ca and P signal intensity than more central dentin areas (Fig 6B) and can thus not be considered indicative of a hypermineralized surface layer of enameloid. The phenomenon may rather represent an artifact due to a slight charging of the specimen.

**Table 1:** Calcium (Ca) and phosphorus (P) concentrations (normalized values) obtained by SEM-EDS for dentin and bone of attachment (eight measurement spots each, for location see Fig 7) of a left first dentary tooth of *Anoplogaster cornuta* and Ca/P ratios for the two tissues. *P*-values (two-tailed) are from unpaired t-tests.

	Dentin (*n* = 8)		Bone of attachment (*n* = 8)		*P*-value
	Mean	SD	Mean	SD	
Ca (wt%)	23.48	0.53	22.38	0.61	0.0018
P (wt%)	10.13	0.19	9.13	0.19	< 0.0001
Ca/P ratio	2.32	0.03	2.45	0.06	< 0.0001

wt% = weight percent, SD = standard deviation.

For individual values see S1 Table.

### Pharyngeal teeth

**Fig 9:**
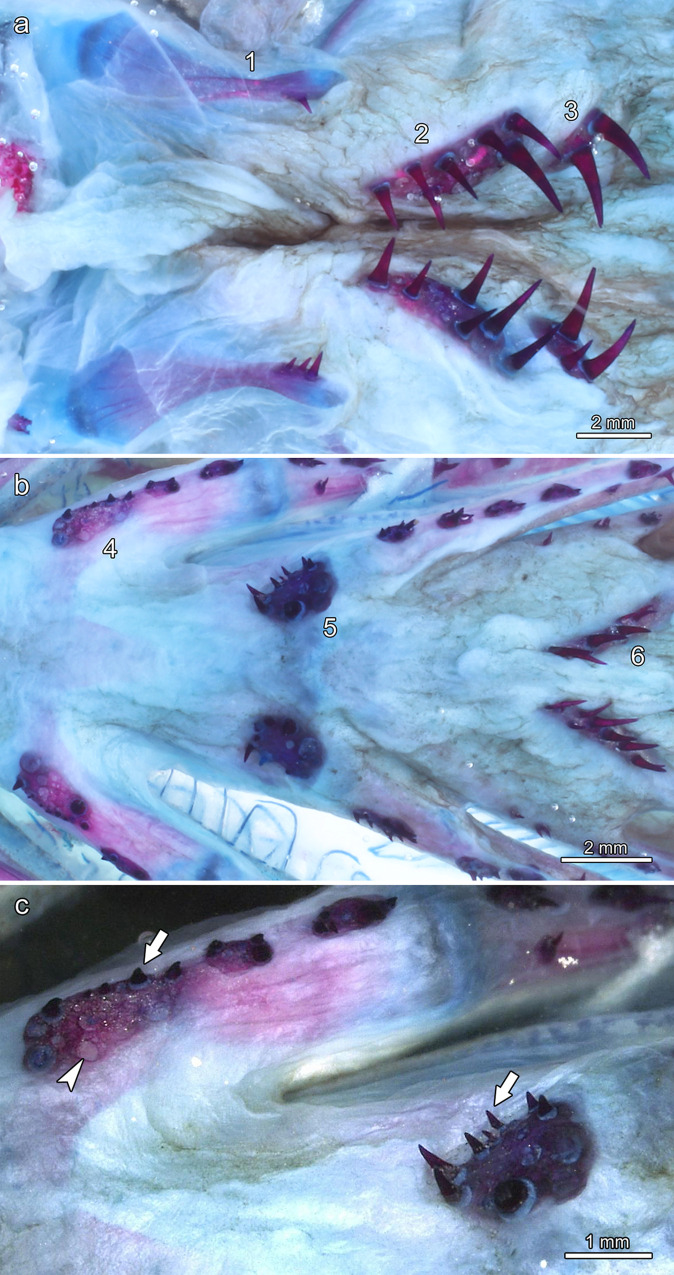
Tooth fields in the dorsal and ventral oropharynx of *Anoplogaster cornuta*, cleared and stained (Alizarin red S, alcian blue 8 GX) specimen. **(a)** In the roof of the oropharynx, paired tooth fields are located on the dermopalatines (1), the ectopterygoids (2) and the entopterygoids (3). Note that the teeth on the posterior tooth fields are larger than those on the dermopalatines. **(b)** In the floor of the oropharynx, paired tooth fields are present on the pharyngobranchials of the second (4) and third (5) branchial arches and on the ceratobranchials of the fifth branchial arch (6). **(c)** Higher magnification of tooth fields on the pharyngobranchials. Note presence of small teeth (arrows) diagnosed as replacement teeth and of empty tooth loci (one marked by arrowhead).

**Fig 10:**
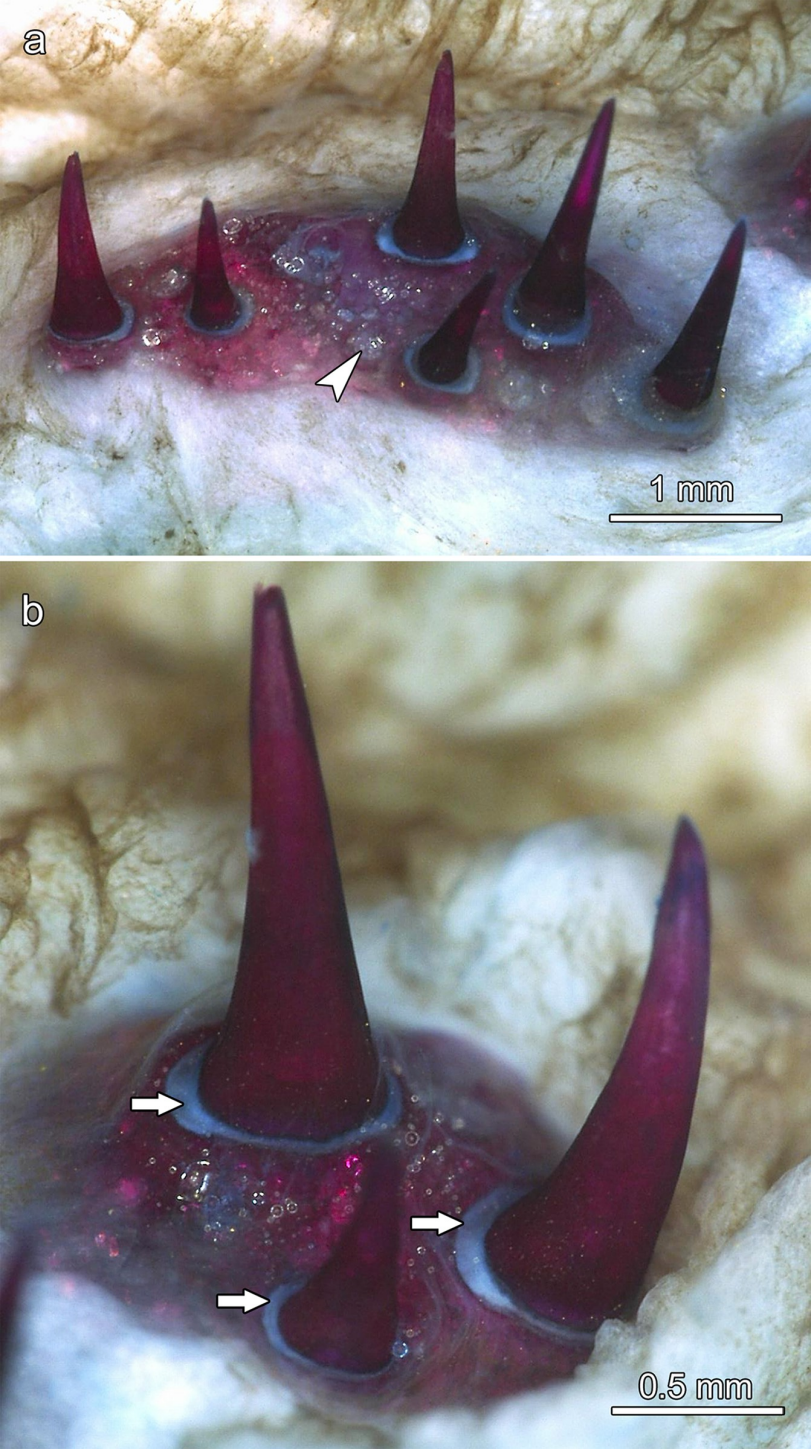
Tooth fields in the dorsal oropharynx of *Anoplogaster cornuta*, cleared and stained (Alizarin red S, alcian blue 8 GX) specimen. **(a)** Tooth field on the right ectopterygoid, note empty tooth locus (arrowhead). **(b) T**ooth field on the right entopterygoid. Note ring-like collagenous tissue (arrows in b) connecting the tooth base to the dentigerous bone.

**Fig 11:**
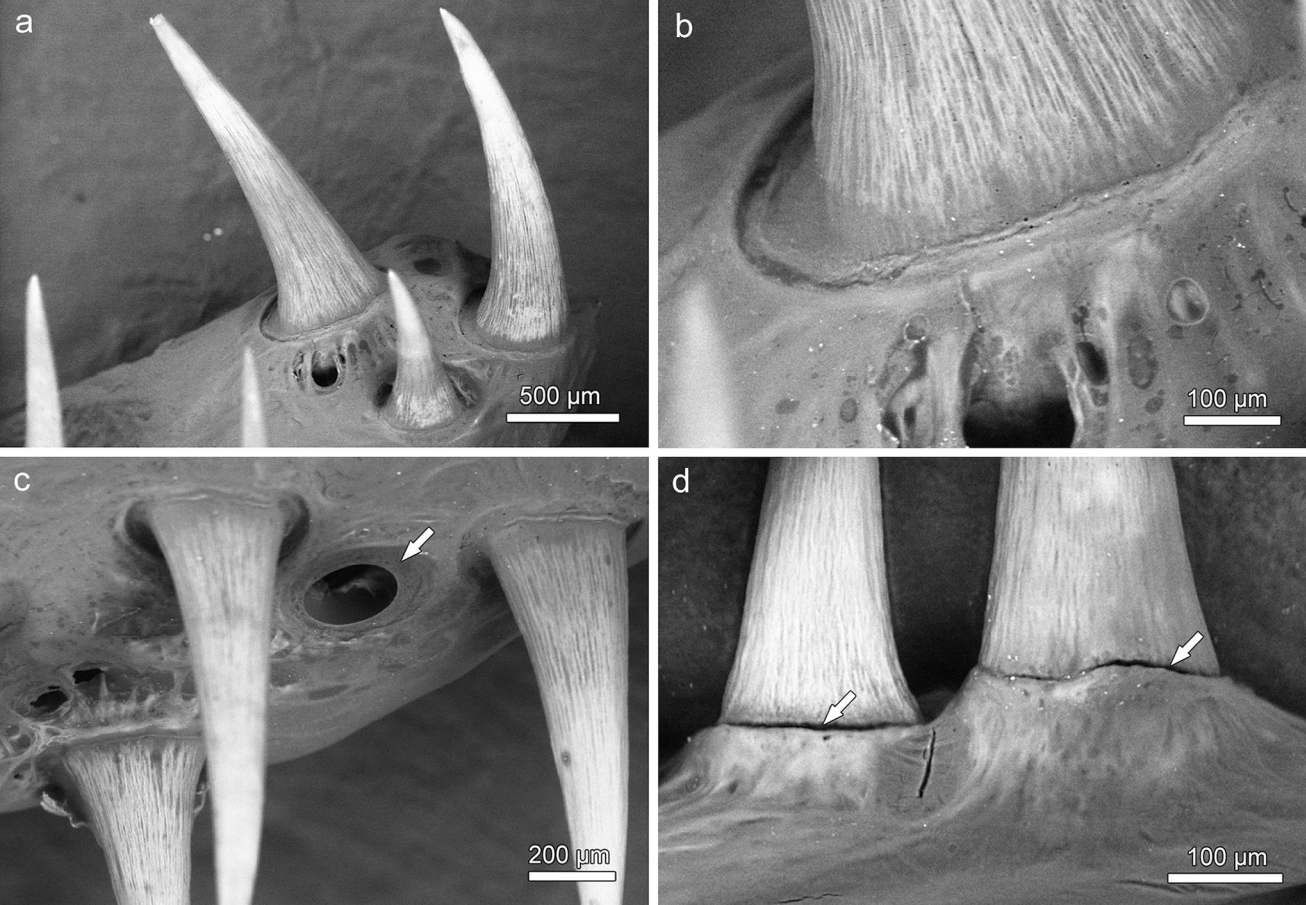
SEM-BSE images of tooth-bearing bones in the dorsal oropharynx of *Anoplogaster cornuta*, macerated specimen, anterior to left of images. **(a)** Teeth from the right ectopterygoid. The small tooth is regarded as a recently established replacement tooth. **(b)** Detail of a tooth base on the right ectopterygoid. The tooth is slightly lowered into the underlying bone cavity. **(c)** Teeth on the left entopterygoid. Note empty tooth position (arrow) with ring-like collagenous attachment structure still in place. **(d)** Pharyngeal teeth on the right dermopalatine attached to a bony socket. Note narrow clefts (arrows) previously occupied by a collagenous attachment structure.

In the roof and floor of the central oropharynx, a total of six paired tooth fields were present (Fig 9A and 9B). The teeth situated on these bones were monocuspid and slightly recurved, with the largest teeth occurring on the most posteriorly located bony plates. In the dorsal oropharynx, paired tooth fields were present on the dermopalatines and the ecto- and entopterygoids (Fig 9A). In the ventral oropharynx, paired tooth fields were present on the pharyngobranchials of the second and third branchial arch, while a third paired tooth field was located on the ceratobranchials of the fifth branchial arch (Fig 9B). In places, small replacement teeth and empty tooth loci were discernible in the pharyngeal tooth fields (Figs 9C, 10A, 11A and 11C).

**Fig 12:**
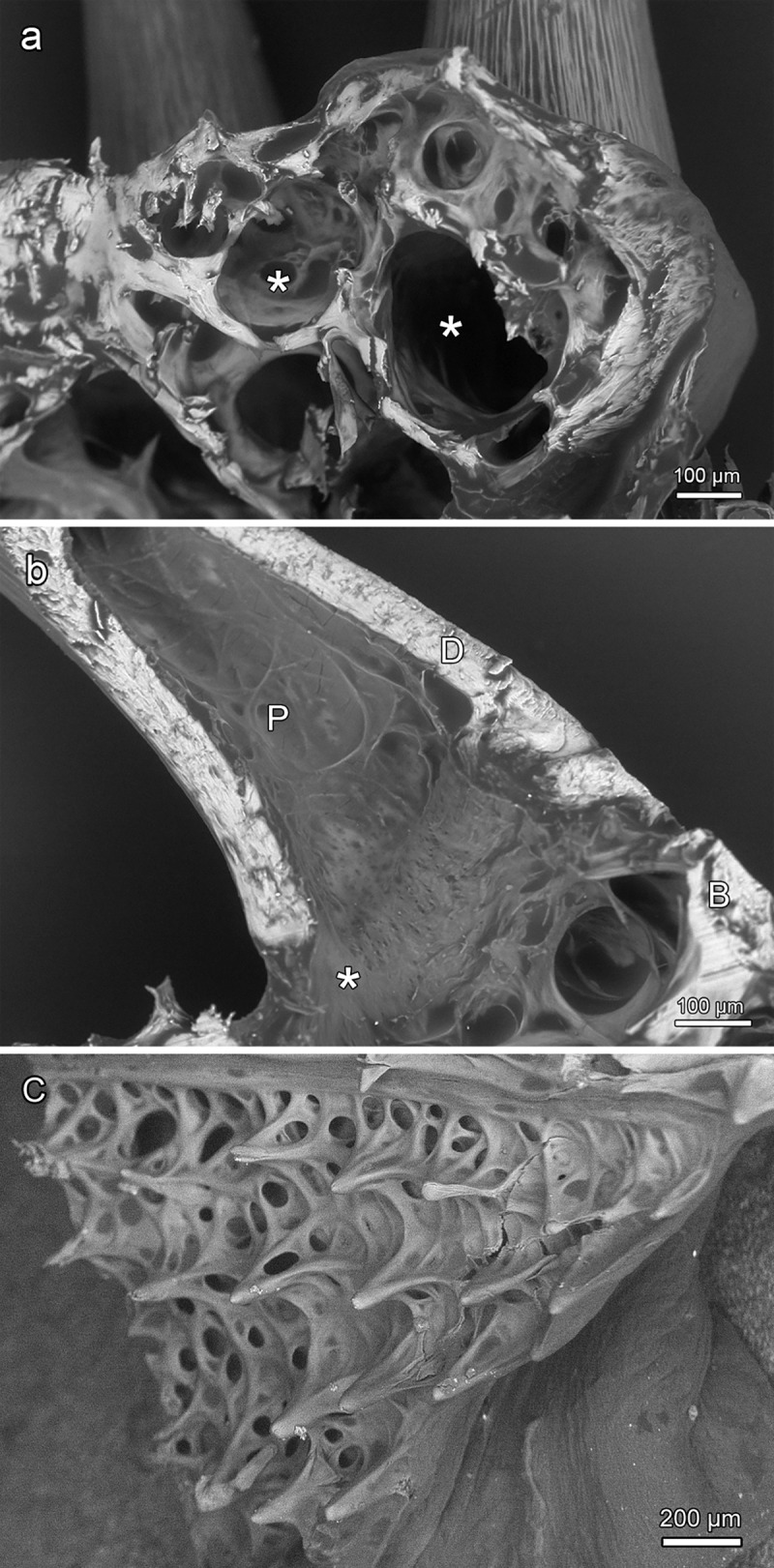
**SEM-BSE images of teeth and dentigerous bone (a, b) and bony projections (odontoids) (c) in the oropharynx of *Anoplogaster cornuta*. (a)** Multiple cavities (asterisks) are present in the entopterygoid. **(b)** Broad connection between pulp cavity (P) and cavities in the dentigerous bone (B) (entopterygoid). Note collagenous connection (asterisk) between tooth base and bone. D: Dentin. **(c)** Bony projections (odontoids) emanating from a mesh-like bone in the roof of the oropharynx. Posterior to left of image.

Except for those on the dermopalatines, all teeth present in the central oropharynx exhibited a relatively broad and flat, ring-like collagenous connection between their base and the dentigerous bone (Figs 10A, 10B and 11A–11C). In places where teeth had been lost, their previous attachment site on the dentigerous bone was clearly discernible (Fig 11C). The multiple cavities present in the latter communicated with the pulp spaces of the attached teeth (Fig 12A and 12B). In a longitudinal section through a tooth from the right entopterygoid, the collagenous attachment to the underlying dentigerous bone was well visible (Fig 12B). The hammock-like collagenous attachment between tooth base and dentigerous bone is considered to allow both lateral mobility and a certain depression of the tooth. Following (partial) enzymatic digestion (and weakening) of the collagenous attachment, single teeth had become slightly lowered into the underlying bony cavities (Fig 11A and 11B). A similar, however, reversible movement is considered to occur intravitally when these teeth are exposed to mechanical loading.

The teeth on the dermopalatines were attached by a collagenous tissue to bony sockets that protruded from the dentigerous bone. In macerated specimens, the area of the fibrous connection appeared as a narrow cleft (Fig 11D).

Bony plates exhibiting numerous pointed projections were present in the dorsal oropharynx lateral to the anterior portion of the parasphenoid. They were arranged in rows and continuous with the underlying bone without a detectable boundary or difference in mineralization (Fig 12C). The projections are thus not considered to represent true teeth but are classified as tooth-like bony structures (odontoids).

**Fig 13:**
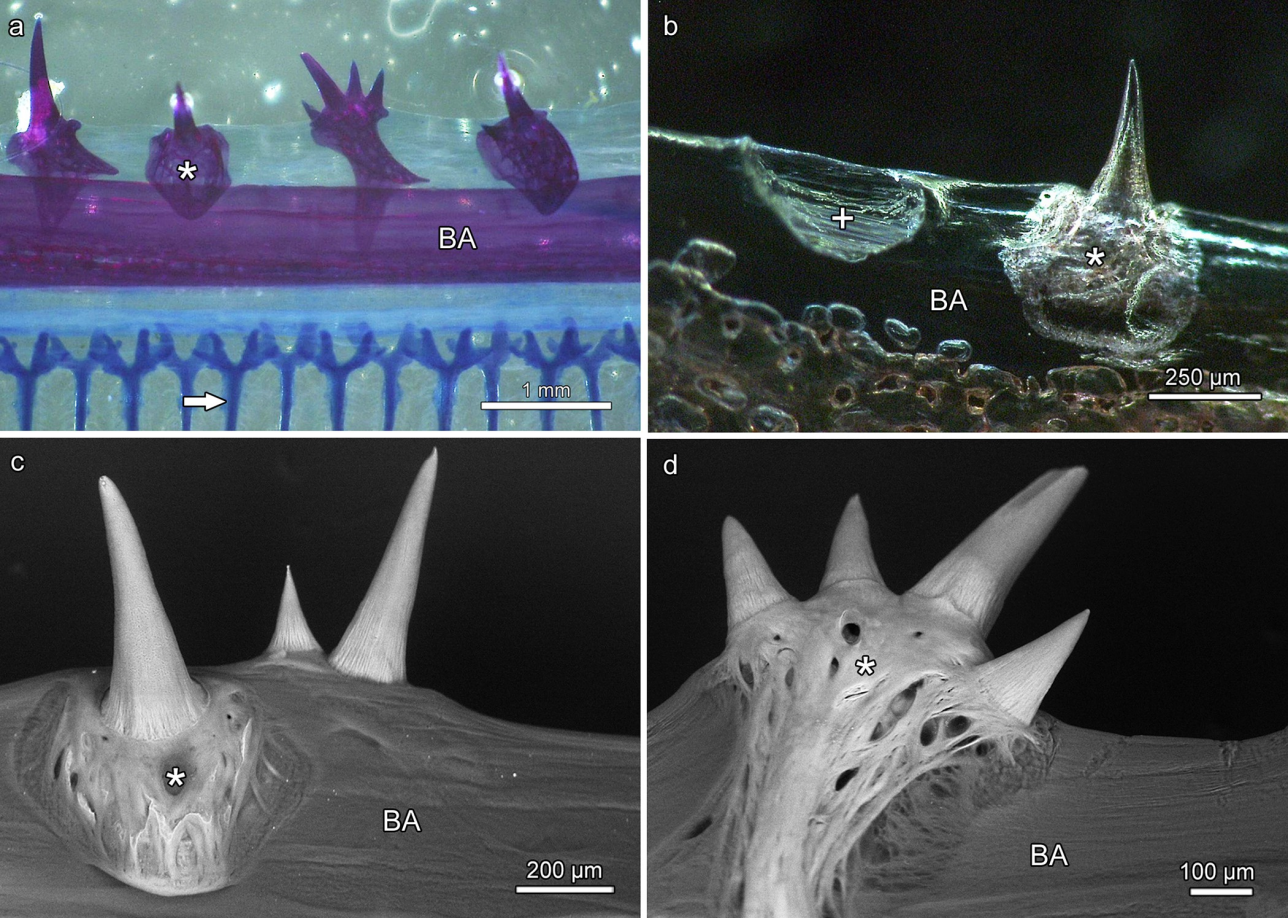
Teeth on the branchial arches of *Anoplogaster cornuta*. **(a)** Single or multiple monocuspid teeth are attached to trabecular bony plates (asterisk) located on a branchial arch (BA). Arrow: Gill filament. Cleared and stained (Alizarin red S, alcian blue 8 GX) specimen. **(b)** Bony plate (asterisk) with single attached tooth and cup-like notch (+) on a branchial arch (BA) where a tooth-bearing bony plate has been lost. Unstained, macerated specimen. **(c) and (d)** SEM-BSE images of single (c) or multiple (d) monocuspid teeth attached to bony plates (asterisks) with trabecular architecture. Note ring-like seams at the attachment sites of the teeth on the bony plates. The bony plates are fixed by collagen fibers to the branchial arches (BA).

Numerous small teeth were present on the branchial arches (Fig 13A). Except for the most posterior branchial arch, which possessed only a single tooth row, two rows of teeth were present on each branchial element. Typically, the teeth of the outer row were larger than those of the inner row. Single or multiple monocuspid teeth were firmly attached to bony plates that exhibited a trabecular architecture (Fig 13A–13D). A ring-like seam marked the attachment sites of the teeth to the dentigerous bone plates that themselves were fixed to the underlying branchial arch by collagenous fibers (Fig 13C and 13D). In places where dentigerous bone plates (plus teeth) had been lost, a shallow cup-like notch was present on the branchial arch (Fig 13B).

## Discussion

This is the first study providing detailed information on the structure of jaw and pharyngeal teeth and their attachment modes in adult *Anoplogaster cornuta*. The widely spaced teeth in the upper and lower oral jaws of *Anoplogaster cornuta* were firmly attached to the respective jawbones (premaxilla, dentary) via a bone of attachment and non-depressible. This attachment mode corresponds to Type 1 of Fink’s classification [7], and our findings are in full accordance with his statement on the attachment mode of the oral jaw teeth in *Anoplogaster cornuta*. Fink [7] considered this type of tooth attachment to represent the plesiomorphic condition in actinopterygians. Functionally, the firmly ankylosed jaw teeth of *Anoplogaster cornuta* may form a kind of cage that prevents captured larger prey from escaping once the mouth is partly closed. A similar function has previously also been assumed for the large jaw teeth of other predatory deep-sea fishes [14–16]. The collar of mineralized collagen fibers connecting the basal portions of the jaw teeth and the bone of attachment in *Anoplogaster cornuta* is considered to provide additional mechanical stability to the teeth during the catching of larger prey.

A further feature that the large oral jaw teeth of *Anoplogaster cornuta* share with those of other predatory deep-sea fishes, e.g., viperfish, *Chauliodus sloani*, and dragon fish, *Aristostomias scintillans*is [14,16], is their transparency. Both the dragonfish and the viperfish use bioluminescence by photophores to attract potential prey in their dark deep-sea environment. It has been suggested that the transparency of their teeth increases predatory success as it renders the teeth of the opened mouth indiscernible against the blackness of the fish body and the background darkness of the sea [16]. It is, however, unlikely that this explanation also holds for *Anoplogaster cornuta*, as this species lacks photophores [6] In dragonfish teeth, it has been demonstrated that the optical transparency of the dental hard tissues is related to a special nanoscale structure that causes a much-reduced Rayleigh light scattering of these tissues [16].

We found no evidence for a replacement of the large teeth located in the premaxillary and the dentary of the two analyzed individuals. In teleosts, replacement teeth can either be formed in the soft tissue outside the tooth-bearing bones (extraosseous development) or within bony crypts beneath their functional predecessors (intraosseous development) [17]. Extraosseus development of replacement teeth is seen as a plesiomorphic, intraosseaus development as a derived trait that has developed independently in at least three teleost clades [17]. Incoming replacements for the large oral jaw teeth of *Anoplogaster cornuta* were not observed in the cleared and stained specimen. Also, no signs of resorption indicating beginning of tooth loss were recorded at the bases of the functional oral jaw teeth. This could indicate that in adult individuals of this species a replacement of oral jaw teeth does either not take place at all or occurs only at very extended intervals. By contrast, in *Chauliodus sloani*, a deep-sea fish that also possesses long, conspicuous jaw teeth, Tchernavin [18] reported the presence of replacement teeth located close to the functional premaxillary and dentary teeth.

In the rainbow trout (*Oncorhynchus mykiss*), the time between the first appearance of a tooth in the mouth and its replacement by a successor, was established on average with 8 weeks in small individuals (body length 12–15 cm) and 12–14 weeks in larger specimen (body length 20–23 cm) [19,20]. In the bluefish (*Pomatomus saltatrix*), a pelagic and coastal marine predator with thecodont oral jaw teeth, about half of the tooth loci in the premaxilla and the dentary exhibited functional or eroding teeth, while the other half of the tooth loci was characterized by absent or incoming teeth [21]. This indicates a regular alternate replacement of oral jaw teeth in this species. It seems possible that the limited resource availability in its deep sea habitats and the necessity to secure the cage function of the jaws either precludes or considerably prolongates the replacement of the large acrodont oral jaw teeth of adult *Anoplogaster cornuta*. Further studies addressing the question of replacement of oral jaw teeth in this species are necessary to clarify this issue.

Our findings on the distribution of pharyngeal jaw teeth in *Anoplogaster cornuta* matches previous findings in this species [22] and other teleosts [23]. In contrast to the oral jaw teeth, the pharyngeal teeth of *Anoplogaster cornuta* are replaced as is indicated by the presence of empty tooth loci and small, newly established teeth. The pharyngeal teeth exhibit a ring-like collagenous attachment to their supporting (dentigerous) bones, a condition that allows a marked mobility of the teeth and may facilitate intrapharyngeal prey transport and processing [4,24]. Intrapharyngeal prey transport is further facilitated by the fact that several attached muscles enable a high mobility of the tooth bearing bones [24].

As has been demonstrated in the eel, *Synaphobranchus kaupi* [7], the presence of such a flexible collagenous ring at their base allows for a certain depression of the teeth even in the absence of a specialized hinge mechanism. Due to the flexibility of the fibrous attachment, the depressed teeth will return to their previous position once released from mechanical load, thereby exercising a cushioning effect during feeding. Fink [7] regarded this type of tooth attachment to occur in non-euteleosteans, which lack the specialized depression mechanism provided by hinged teeth. Our findings in *Anoplogaster cornuta*, however, reveal that this mechanism of tooth attachment and depression also occurs in members of the Euteleostei [1]. Our findings on the attachment modes of pharyngeal teeth in *Anoplogaster cornuta* are therefore at variance with those of Fink [7] who reported a posterior-hinged attachment mode (his Type 4) for all pharyngeal teeth in this species.

In *Anoplogaster cornuta*, numerous small teeth were present on bony plates located on the branchial arches. Single or multiple monocuspid teeth were firmly attached to these bony plates that themselves were fixed to the respective branchial arch elements by collagenous fibers. The function of these teeth in deep-sea fishes has been previously analyzed [25]. These authors argue that the sparse food supply in the deep-sea requires a tightness of the pharyngeal basket that allows the retention of even small food items. In addition to well-developed gill rakers or gill sieves, spines or teeth located on the branchial arches may serve this purpose. In the analyzed mesopelagic and bathypelagic fishes, Ebeling and Cailliet [25] found pharyngeal baskets equipped with additional spines and/or teeth that allow to retain and concentrate small prey items down to about 1,0 mm in diameter. In line with this view, we assume that retention of small prey items is the primary function also of the numerous small teeth present on the branchial arches of *Anoplogaster cornuta*. The function of the bony projections (odontoids) in the dorsal oropharynx of *Anoplogaster cornuta* remains to be elucidated. They may play a role in the firm holding of larger prey items.

Observations by light and scanning-electron microscopy revealed that the teeth of *Anoplogaster cornuta* consisted entirely of orthodentin, whose dentinal tubules were typically occluded near the tooth surface. A hypermineralized enameloid cap, which covers the dentin in many actinopterygian species [4,26,27], was not recorded. Occlusion of dentinal tubules may be considered a protective mechanism in the absence of enameloid. The latter could indicate that in *Anoplogaster cornuta* inactivation of the matrix secretory calcium-binding phosphoprotein 5 (*SCPP5*) gene has occurred, which in actinopterygians regulate the formation of hypermineralized enameloid [28,29]. It would be interesting to analyze if this condition is already present in the teeth of larvae or if its occurrence is restricted to adult individuals.

A juxtapulpal (inner) dentin layer characterized by a reduced density, and partially a complete absence, of dentinal tubules was found in the oral jaw teeth of *Anoplogaster cornuta*. Zones of juxtapulpal atubular dentin have thus far only rarely been described in fish teeth. Shellis and Poole [30] reported their occurrence in *Latimeria chalumnae*, and Berkovitz and Shellis [4] illustrated another example in the tooth of a thornback ray (*Raja clavate*). The latter authors hypothesized that the zone of atubular dentin may represent a form of secondary or tertiary dentin present only in mature fish teeth. Whether the inner dentin zone with reduced tubular density observed in the oral jaw teeth of *Anoplogaster cornuta* represents secondary or tertiary dentin, as is assumed to be the case in *Latimeria* and *Raja* [4,30], requires further study.

In the oral jaw teeth of *Anoplogaster cornuta*, the boundary between dentin and bone of attachment was discernible on microscopic inspection, as the bone of attachment lacked the tubular structure of the dentin. SEM-BSE imaging and SEM-EDS measurements demonstrated that the dentin of the teeth of *Anoplogaster cornuta* was more highly mineralized and exhibited a lower Ca/P weight ratio than the bone of attachment. Mean Ca/P ratios of dentin (2.32) and bone of attachment (2.45) were higher than that of stoichiometric hydroxyapatite (2.15) and indicate some substitution (e.g., of phosphate by carbonate) in the mineral [31,32]. The differences in the degree of mineralization and in mineral composition also enable a distinction between the two mineralized tissues that in zebrafish were shown to be produced by different scleroblastic cell types, namely odontoblasts (dentin) and osteoblasts (bone of attachment) [8]. At present it is unclear why, in contrast to the supporting dentigerous bone, osteoblasts become only very rarely entrapped as osteocytes in the bone of attachment. A recent study on mineralizing primary calvarial cell cultures of mice suggested a role of the highly dynamic mode of collagen assembly and maturation in this process [33]. These authors argue that slight differences in collagen organization may decide whether osteoblasts become embedded as osteocytes or not. It remains to be analyzed if this also holds for the difference in the embedding of osteoblast as osteocytes between dentigerous bone and bone of attachment in teleosts. There is consensus that possession of cellular bone is the ancestral condition for teleosts, and acellular bone has been suggested as a synapomorphy of the Euteleostei [34]. In this respect it is interesting to note that in *Anoplogaster cornuta* only the bone of attachment is (largely) anosteocytic, while the dentigerous bones contain osteocyte lacunae.

In summary, our study revealed that two main types of tooth attachment modes are present in *Anoplogaster cornuta*. A firm ankylosis between dentinal tooth shaft and bone of attachment (Type 1 of Fink’s classification) is present in the oral jaw teeth. In contrast, a ring-like fibrous (collageneous) attachment to the underlying bone (Type 2 of Fink’s classification) was found in most of the larger pharyngeal teeth. Our results therefore corroborate previous statements by Fink [5] on the attachment mode of the oral jaw teeth (Type 1) of *Anoplogaster cornuta*, but are at variance with his statement of the presence of a Type 4 attachment mode in the pharyngeal teeth of the species. All teeth in *Anoplogaster cornuta* were monocuspid and composed solely of orthodentin, without a covering cap of enameloid. No evidence for replacement of the large oral jaw teeth was found in the analyzed adult specimen of *Anoplogaster cornuta*. Further studies involving larval and adult individuals are needed to clarify the question of tooth replacement and the lack of enameloid in this species.

## Supporting information

S1 Fig Horizontal section (polished) through the basal shaft of a left first dentary tooth of *Anoplogaster cornuta*. **(a)** SEM-BSE image showing corrugation of the tooth surface and smooth appearance of the pulpal dentin surface. Posterior to top, lateral to left of image. Position of EDS line profile indicated by green line. PC: Pulp cavity **(b)** SEM-EDS line profile showing variation of calcium (Ca) and phosphorus (P) concentrations (relative values) across the medial dentinal wall. Asterisk: Pulp cavity.
(TIF)

S1 Table Results of SEM-EDS multipoint analysis of dentin and bone of attachment in a left first dentary tooth of *Anoplogaster cornuta*. (DOCX)
