# Supplementary figures and images for: Dental structure and tooth attachment modes in the common fangtooth Anoplogaster cornuta (Valenciennes, 1833) (Actinopterygii; Trachichthyiformes; Anoplogastridae)
Source: PLoS One. 2022 Aug 12;17(8):e0272860. doi: 10.1371/journal.pone.0272860 (PMC9374257; doi:10.1371/journal.pone.0272860)

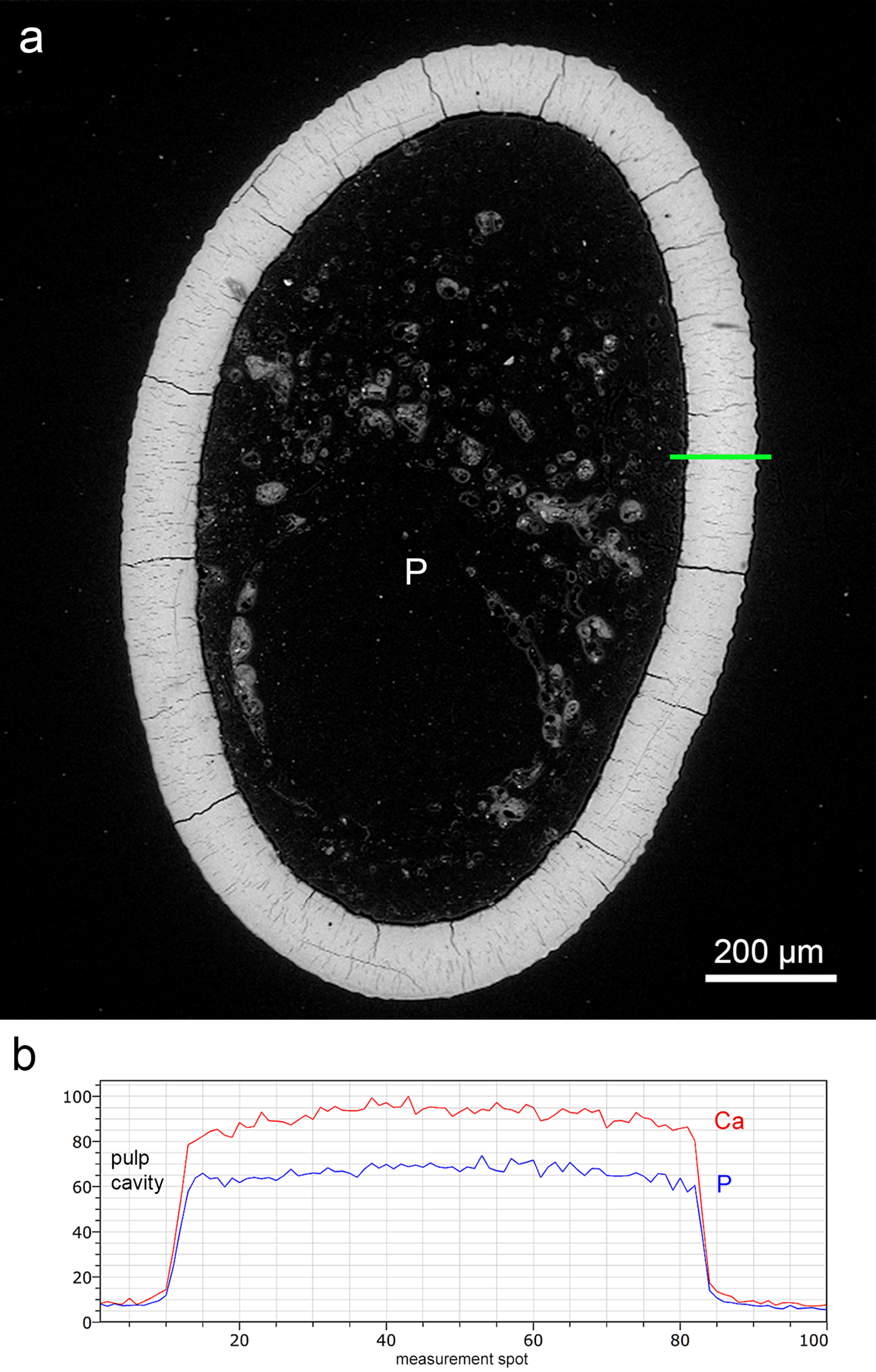

Supplement: S1 Fig — (a) SEM-BSE image showing corrugation of the tooth surface and smooth appearance of the pulpal dentin surface. Posterior to top, lateral to left of image. Position of EDS line profile indicated by green line. PC: Pulp cavity (b) SEM-EDS line profile showing variation of calcium (Ca) and phosphorus (P) concentrations (relative values) across the medial dentinal wall. Asterisk: Pulp cavity. (TIF) [file pone.0272860.s001.tif]
