# Supplementary material for: Dental structure and tooth attachment modes in the common fangtooth Anoplogaster cornuta (Valenciennes, 1833) (Actinopterygii; Trachichthyiformes; Anoplogastridae)
Source: PLoS One. 2022 Aug 12;17(8):e0272860. doi: 10.1371/journal.pone.0272860 (PMC9374257; doi:10.1371/journal.pone.0272860)
Supplement: S1 Table — (DOCX) [file pone.0272860.s002.docx]

**S2 Table.** Results of SEM-EDS multipoint analysis of dentin and bone of attachment in a left first dentary tooth of *Anoplogaster cornuta*

| **Measurement No.** | **Tissue (Dentin/Bone)** | **Calcium concentration (wt%, normalized)** | **Phosphorus concentration (wt%, normalized)** | **Ca/P weight ratio** |
| --- | --- | --- | --- | --- |
| 1 | Dentin | 23.24 | 10.16 | 2.2874 |
| 2 | Dentin | 23.39 | 9.87 | 2.3698 |
| 3 | Dentin | 24.04 | 10.31 | 2.3317 |
| 4 | Dentin | 23.69 | 10.22 | 2.3180 |
| 5 | Dentin | 24.25 | 10.39 | 2.3340 |
| 6 | Dentin | 22.71 | 9.89 | 2.2963 |
| 7 | Dentin | 23.61 | 10.18 | 2.3193 |
| 8 | Dentin | 22.89 | 9.99 | 2.2913 |
| 9 | Bone | 21.68 | 8.88 | 2.4414 |
| 10 | Bone | 23.47 | 9.15 | 2.5650 |
| 11 | Bone | 22.92 | 9.43 | 2.4305 |
| 12 | Bone | 21.83 | 9.17 | 2.3806 |
| 13 | Bone | 22.67 | 9.09 | 2.4939 |
| 14 | Bone | 22.21 | 9.26 | 2.3985 |
| 15 | Bone | 21.90 | 8.86 | 2.4718 |
| 16 | Bone | 22.37 | 9.22 | 2.4262 |
